# Reply to Dumke, C. Comment on “Fan et al. Efficacy of Ingesting an Oral Rehydration Solution after Exercise on Fluid Balance and Endurance Performance. *Nutrients* 2020, *12*, 3826”

**DOI:** 10.3390/nu13093215

**Published:** 2021-09-16

**Authors:** Jason Kai Wei Lee, Priscilla Weiping Fan, Stephen F. Burns

**Affiliations:** 1 Human Potential Translational Research Programme, Yong Loo Lin School of Medicine, National University of Singapore, Singapore S119228, Singapore; 2 Department of Physiology, Yong Loo Lin School of Medicine, National University of Singapore, Singapore S117593, Singapore; 3 Global Asia Institute, National University of Singapore, Singapore S119076, Singapore; 4 N.1 Institute for Health, National University of Singapore, Singapore S117456, Singapore; 5 Institute for Digital Medicine, National University of Singapore, Singapore S117456, Singapore; 6 DSO National Laboratories, Defence Medical and Environmental Research Institute, Singapore S117510, Singapore; fweiping@dso.org.sg; 7 National Institute of Education, Nanyang Technological University, Singapore S637616, Singapore; stephen.burns@nie.edu.sg

We would like to thank Dr. Charles Dumke for taking interest in our recent publication [1]. In our study, we aimed to investigate the rehydration efficacy of ingesting an oral rehydration solution during post-exercise recovery followed by a pre-load time trial performance. Dr. Dumke provided comments about the presentation of data, extrapolation of results and external validity.

Firstly, Dr. Dumke mentioned an error in the presentation of fluid retention data in Figure 1b. This was an oversight on our part and we have submitted a correction.

Secondly, it was pointed out that one should not extrapolate the effectiveness of oral rehydration solutions from promoting rehydration after diarrhoea to during exercise in the heat. We agree and no such extrapolation was made in our paper. It is noteworthy that, while Dr. Dumke’s previous study investigated fluid retention during exercise [2], our study was designed to represent individuals who may have to execute two exercise sessions within a day. Therefore, we were interested in fluid retention during a post-exercise recovery phase. That may explain the difference in the fluid retention results between the two studies [1,2].

Lastly, Dr. Dumke questioned the external validity of the current study in the case of workers and athletes on the field. While we argued that our design “mimicked the lifestyle of elite endurance athletes and soldiers where two bouts of exercises can often be completed within a single day” [1], there is validity in that the extra fluid retention is promoted only “in the absence of ingestion of any other nutrition” within 5 h of passive recovery. Nonetheless, our study methodology to deduce hydration efficacy was based on fluid retention following post exercise fluid replenishment is in line with several post exercise rehydration studies [3,4,5]. Therefore, this does not invalidate the experimental demonstration of the effectiveness of the oral rehydration solution.
